# Scaling up contraceptives use in the division with lowest contraceptives use in Bangladesh: sources, methods, and determinants

**DOI:** 10.1186/s40748-017-0049-x

**Published:** 2017-06-06

**Authors:** Gulam Muhammed Al Kibria, Vanessa Burrowes, Sharmin Majumder, Atia Sharmeen, Rifath Ara Alam Barsha, Shakir Hossen

**Affiliations:** 1 Johns Hopkins Bloomberg School of Public Health, Baltimore, MD USA; 2 Faculty of Public Health, Mahidol University, Salaya, Thailand; 3 Shimantik, Sylhet, Bangladesh; 4 Department of Pediatrics, Sylhet M. A. G. Osmani Medical College Hospital, Sylhet, Bangladesh

**Keywords:** Contraceptive prevalence rate, Contraceptives, Family Planning 2020, Distributions, Determinants, Methods, Sylhet, Bangladesh

## Abstract

**Background:**

Total fertility rate (TFR) is high and at a static level for the last two decades in Bangladesh. Reduction of fertility by increasing contraceptives use could reduce maternal and neonatal mortality. To achieve the targeted contraceptive prevalence rate (CPR) of Family Planning 2020 (FP2020) Initiative, it is important to increase CPR in all regions of the country. However, it is lower in Sylhet Division compared to other divisions in Bangladesh. This study looked into the methods, source and determinants of contraceptives use in this division.

**Methods:**

Data from the Bangladesh Demographic and Health Survey 2014 (BDHS 2014) were analyzed. After reporting the sources of obtaining contraceptives and choice of methods, distributions of contraceptives use were reported by selected characteristics. Logistic regression was applied to calculate the odds ratios.

**Results:**

A total of 599 women were analyzed. CPR was lower in rural areas compared to urban areas, 45.4% and 58.5%, respectively. The majority of the women received services from governmental sectors. The birth control pill was the most common contraceptive method. The proportion of women using long-acting permanent methods was low (<10%) in both urban and rural areas.

In the multivariate analyses, number of alive children (adjusted odds ratio (AOR) of ≥5 children: 1.6, 95% confidence interval (CI): 1.1–2.2), presence of a male child (AOR: 1.7; 95% CI: 1.1–2.6), higher education level of the husband (AOR: 1.7; 95% CI: 1.1–2.6), receiving a visit from a family planning worker (AOR: 2.4; 95% CI: 1.6–3.4) and membership in a non-governmental organization (AOR:1.4, 95% CI: 1.1–1.8) were positively associated with contraceptives use in Sylhet after controlling for age, education level and other contextual factors. Conversely, rural women had the lower likelihood of using contraceptives (AOR: 0.6; 95% CI: 0.4–0.8) than urban women. Women’s education level and religion were not statistically significant.

**Conclusions:**

A comprehensive strategy is required for this division to address multiple factors which simultaneously influence contraceptives use. In addition to more awareness programs to increase contraceptives use, providing contraceptive distribution services through family planning workers, involving women with non-governmental organizations and prioritizing rural areas could increase contraceptives use in Sylhet Division.

## Plain English Summary

In Bangladesh, total fertility rate (TFR) is high and at a static level for the last two decades. Increasing use of contraceptives could reduce maternal and neonatal mortality. Within this country, the contraceptives prevalence rate (CPR) is lowest and TFR is highest in Sylhet Division. This study was conducted to investigate the methods, sources and determinants of contraceptives use in the Sylhet Division.

Cross-sectional and secondary data from the Bangladesh Demographic and Health Survey 2014 (BDHS 2014) were analyzed. CPR was lower in rural areas compared to urban areas. The birth control pill was the most common contraceptive method. The proportion of women using long-acting permanent methods was found to be lower in both urban and rural areas.

After controlling for age, education level and other contextual variables, factors which were positively associated contraceptives use are: number of alive children, the presence of a male child, higher education level of the husband, receiving a visit from a family planning worker and membership in a non-governmental organization. Rural women had a lower likelihood of using contraceptives than the urban women.

A simultaneous significance of several factors indicates that a comprehensive strategy is required to address multiple factors. Conducting more awareness programs, providing contraceptive distribution services through family planning workers, involving women with non-governmental organizations and prioritizing rural areas were recommended to increase contraceptives use in that region of the country.

## Background

Despite global reduction of fertility, total fertility rate (TFR) remains high in several countries including Bangladesh [1]. TFR declined substantially from 6.3 in 1975 to 3.4 per woman in 1994. However, the progress was slow over a period of 20 years, from 3.4 in 1994 to 2.3 per woman in 2014. In addition to high fertility, maternal and neonatal mortality are high in Bangladesh [2]. Family planning could reduce maternal and neonatal mortality by preventing pregnancy among adolescents and prolonging birth intervals among adult females [3–8]. Scaling up contraceptives use could help meet the maternal and neonatal mortality targets of the United Nations’ Sustainable Development Goals (SDGs) [9].

To prioritize family planning, the Ministry of Health and Family Welfare (MOHFW) of the Government of Bangladesh set targets to increase contraceptive use prevalence rate (CPR) from current estimates of 62% to 70% and to reduce TFR to 2.0 as a part of Family Planning 2020 (FP2020) Initiative. Increasing CPR in low-performing divisions (the largest administrative unit of the country) and increasing current CPR to 60% coverage are also components of the plan [10], since it is not possible to achieve the target without increasing contraceptives use in the divisions where CPR is lower. Sylhet Division, located in the northeastern part of Bangladesh, has a consistently lower CPR compared to the rest of the country with an estimated CPR of 48% in 2014. Within the last 18 years, the CPR increased slowly by an annual increment of 1.5% [2]. However, the coverage rate needs to be accelerated to achieve the targets of FP2020.

Due to the slow progress of increasing CPR and reducing fertility over the last 20 years, a clear, focused, and evidence-based approach is needed to increase contraceptives use in this part of Bangladesh. Identifying sources to receive contraceptives services, patterns or methods of contraceptive use, and factors which influence contraceptives use are necessary to formulate a strategy. Earlier studies in Bangladesh found that contextual, fertility and individual factors simultaneously affect contraceptives use, and this use is significantly lower among rural women compared to urban women [11–13]. Although a large number of studies have looked into contraceptives use in Bangladesh, no studies have investigated sources, methods and determinants related to contraceptives use particularly in this division. In this study, we used a representative dataset to identify sources, patterns, and factors associated with contraceptives use in Sylhet division of Bangladesh. The findings can then be used to inform policymakers and researchers to target for intervention and strengthen efforts to scale up the rate of contraceptive use in this area. Findings from our study could also be used to inform policymakers and researchers of other countries with similar scenarios.

## Methods

### Ethics statement

Ethical approval for this study was not required as the data were available for use upon approval. We obtained permission to use the data from the ICF International, Rockville, Maryland, USA in January 2017.

### Data source

We analyzed data collected from the Bangladesh Demographic and Health Survey (2014 BDHS). Mitra and Associates implemented the survey from June to November 2014. Details of the survey have been described previously [2]. A household questionnaire, a women’s questionnaire, and a community questionnaire were used in the 2014 BDHS. The women’s questionnaire was used to collect information from ever-married women aged 15–49 years. A total of 164 field workers were recruited based on experience and education level and were trained to conduct the verbal interview [2].

In addition to contraceptives use, women were asked questions on the following topics: demographic characteristics (e.g., age, education, religion, media exposure), reproductive history, antenatal care, delivery, postnatal care, and newborn care, breastfeeding and infant feeding practices, child immunizations and illnesses, marriage, fertility preferences, husband’s background and respondent’s work, awareness of AIDS and awareness other sexually transmitted diseases [2].

### Sample design

The 2014 BDHS sample was nationally representative of women across the country. The survey used a sampling frame from the list of enumeration areas (EAs) of the 2011 Population and Housing Census of the People’s Republic of Bangladesh, which was provided by the Bangladesh Bureau of Statistics (BBS) [14].

The survey was based on a two-stage stratified sample of households. In the first stage, 600 EAs were selected with a statistical probability proportional to the EA size, with a resulting 207 EAs in urban areas and 393 in rural areas. A complete household listing operation was then carried out in all of the selected EAs to provide a sampling frame for the second-stage selection of households. In the second stage of sampling, a systematic sample of 30 households on average was selected per EA to provide statistically reliable estimates of key demographic and health variables for the country as a whole, for urban and rural areas separately, and for each of the seven individual divisions. With this design, the survey selected 18,000 residential households which were expected to result in completed interviews with about 18,000 ever-married women [2].

The weighted distribution of urban-rural households in the survey was based on the urban-rural distribution in the 2011 population census of the country; a modified urban-rural household distribution was reflected by adjusting the sample weights. The adjustment in the 2014 BDHS sampling weight generated a revised urban-rural population distribution and any significant differences in the overall survey indicators were not expected [2].

### Statistical analyses

In the BDHS survey, a current user of contraceptives was defined as currently married women who report that they or their husbands were using any family planning methods at the time of the survey. We selected potential predictors based on published reports and our available data.

Stata 13.0 (Stata Corp, College Station, TX) was used for all data analyses in the study [15]. To allow for the adjustments of the cluster sampling design used in this survey, the ‘svy’ command was used in Stata. Weighted frequency was calculated for selected variables.

After reporting contraceptive usage methods, the sources of contraceptives were reported. Then simple and multiple logistic regressions were used to calculate the crude (unadjusted) odds ratios (CORs) and adjusted odds ratios (AORs) of contraceptives use with selected variables, respectively. Odds ratios (ORs) with 95% confidence intervals (CIs) and significance level were calculated. Continuous and discrete variables were converted into categorical variables for logistic regression analyses. After calculating the CORs, variables with a predetermined significance level (*p* < 0.2) were included in the multivariate analysis as this significance level was considered to be sufficient to adjust for statistical confounders [16]. Variance inflation factors (VIFs) were assessed to check multicollinearity among variables before incorporation into the multivariate adjustment.

## Results

After applying weighted frequency, 47.8% of the women were using any contraceptives, and 40.9% were using any modern types of contraceptives such as pills, condom, and injections (Table 1). Modern CPR was higher in urban areas than the rural areas. The birth control pill was the most common method used by women in both urban and rural areas, 30.8% and 19.3% use, respectively.

**Table 1:** Distribution of currently married women according to contraceptive methods and place of residence, Sylhet division, Bangladesh

Type of contraceptives	Methods of contraceptives	Urban N (%)	Rural N (%)	Total N (%)
Not using any contraceptives		85 (41.5)	514 (54.5)	599 (52.2)
Modern				
	Pill	63 (30.8)	182 (19.3)	245 (21.4)
	Injections	15 (7.1)	60 (6.3)	75 (6.5)
	Condom	13 (6.3)	32 (3.5)	45 (4.0)
	Female sterilization	12 (5.8)	65 (6.9)	77 (6.7)
	Male sterilization	2 (1.0)	12 (1.3)	14 (1.2)
	IUD	0 (0)	3 (0.3)	3 (0.3)
	Implants	1(0.3)	8 (0.9)	9 (0.8)
Traditional				
	Periodic abstinence	0 (0)	1 (0.1)	65 (5.7)
	Withdrawals	5 (2.7)	9 (1.0)	14 (1.3)

**Fig. 1:**
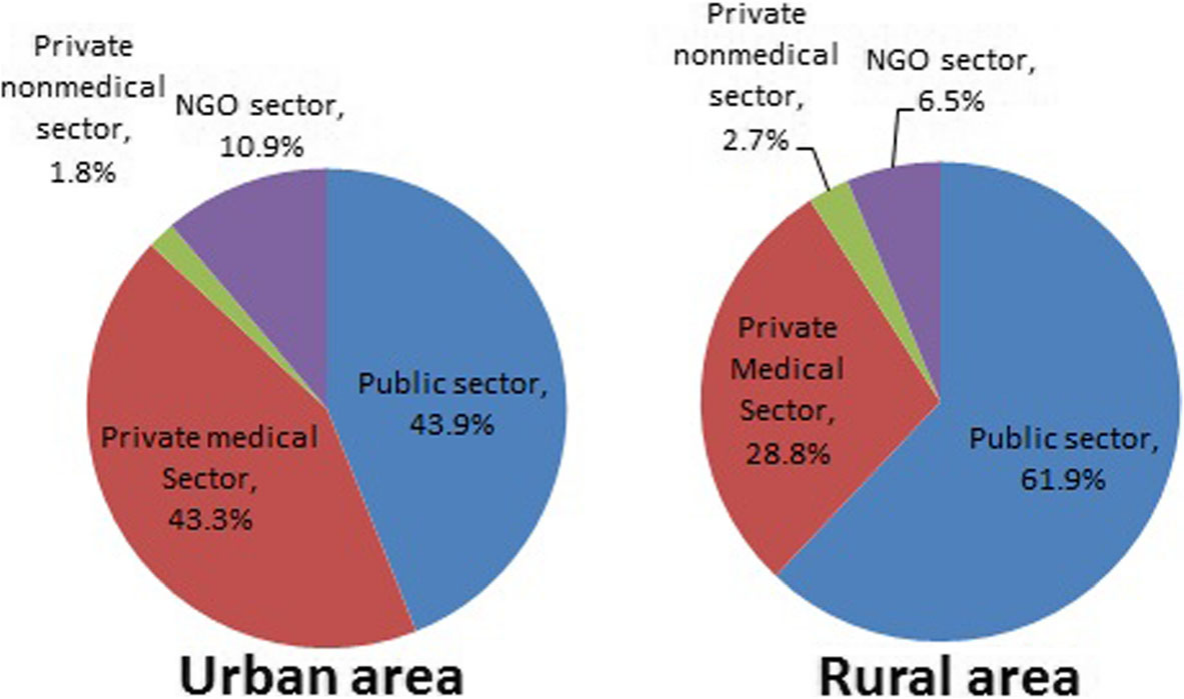
Sources of obtaining modern family planning methods

Sources from which women can obtain modern family planning methods according to the place of residence have been presented in Fig. 1. Overall, the public sector (for instance, district hospital/medical college hospital, maternal and child welfare centers, or upazila health complexes) was the most common source for both urban and rural areas, with 43.9% and 61.9%, respectively. However, an approximately equal percentage (43.3%) of women received contraceptives services from private medical sectors (including private hospitals and clinics, qualified or traditional doctors) in urban areas.

Table 2 presents the distribution of the women according to selected background characteristics and contraceptives use. Numbers and percentages were weighted by an individual sampling of the weight from the BDHS data. Women of the middle age group (25–34 years) were using more contraceptives (58.0%) than the younger (15–24 years) and older women (35–49 years), 34.6% and 49.2%, respectively. CPR was higher among highly educated women and women with highly educated husbands compared to women or their husbands with a lower education level. Only 18.4% of the women received visits by a family planning worker (FPW), and this group was more likely to use contraceptives than the women without any such services, 66.3% and 43.6%, respectively. Similar to FPW visits, women with membership in a non-governmental organization (NGO) were more users of contraceptives (58.8%) than women without any NGO membership (44.7%). CPR of urban women was 58.5% while it was 45.4% among rural women.

**Table 2:** Distribution of currently married women of Sylhet division according to current use of contraceptives and selected background characteristics

Characteristics	Contraceptives use		Total (N(%))^b^
	No (N(%))^a^	Yes (N(%))^a^	
Current Age (years)			
15–24	239 (65.4)	126 (34.6)	365 (31.9)
25–34	178 (42.0)	247 (58.0)	425 (37.0)
35–49	182 (50.8)	175 (49.2)	357 (31.1)
Number of living children			
≤2	363 (62.1)	222 (37.9)	585 (51.0)
3–4	138 (39.3)	214 (60.7)	352 (30.7)
≥5	98 (46.4)	112 (53.6)	210 (18.3)
Have son			
No	230 (71.1)	94 (28.9)	324 (28.3)
Yes	368 (44.7)	455 (55.2)	823 (71.7)
Ever experienced death of a child			
No	462 (52.6)	416 (47.4)	878 (76.6)
Yes	137 (51.0)	132 (49.0)	269 (23.4)
Education level of women			
No education	173 (49.9)	174 (50.1)	347 (30.2)
Primary	222 (54.5)	186 (45.5)	408 (35.6)
Secondary	175 (52.4)	159 (47.6)	334 (29.2)
Higher	28 (49.0)	30 (51.0)	58 (5.0)
Education of husband			
No education	218 (51.3)	207 (48.7)	425 (37.1)
Primary	204 (51.7)	191 (48.3)	395 (34.3)
Secondary	139 (57.8)	102 (42.3)	241 (21.0)
Higher	38 (43.8)	48 (56.2)	86 (7.6)
Working status of women			
No	526 (54.8)	434 (45.2)	960 (83.7)
Yes	73 (38.9)	114 (61.1)	187 (16.3)
Visited by FPW			
No	528 (56.4)	408 (43.6)	936 (81.6)
Yes	71 (33.7)	140 (66.3)	211 (18.4)
NGO membership			
No	494 (55.3)	398 (44.7)	892 (77.8)
Yes	105 (41.2)	150 (58.8)	255 (22.2)
Religion			
Islam	525 (52.7)	470 (47.3)	995 (86.8)
Others	74 (48.8)	78 (51.2)	152 (13.2)
Place of residence			
Urban	85 (41.5)	120 (58.5)	205 (17.9)
Rural	514 (54.6)	428 (45.4)	942 (82.1)
Wealth quintile			
Poorest	168 (54.0)	143 (46.0)	311 (27.2)
Poorer	139 (54.8)	114 (45.2)	253 (22.1)
Middle	95 (47.0)	106 (53.0)	201 (17.5)
Richer	85 (49.2)	87 (50.8)	172 (15.0)
Richest	113 (53.8)	97 (46.2)	210 (18.2)

^a^Row percentage, ^b^Column percentage, *FPW* family planning worker, *NGO* non-governmental organization

Results of simple and multiple logistic regression analyses were presented in Table 3. In the unadjusted analyses, the age of women was a significant predictor of a higher odds of contraceptive use, especially among women aged 25–34 years (COR: 2.6; 95% CI: 2.0–3.5) and 35–49 years (COR: 1.8; 95% CI: 1.3–2.5). The number of children was also a significant predictor. The presence of a male child was the most significant covariate; women who had at least one male child were 3 times more likely to use contraceptives than the women without a male child (COR: 3.0; 95% CI: 2.4–3.8). The education level of women or their husbands and wealth quintile were not significantly associated with contraceptive use. Women receiving a visit by an FPW (COR: 2.5; 95% CI: 1.9–3.5), having NGO membership (COR: 1.8; 95% CI: 1.4–2.2) and employed women (COR: 1.9; 95% CI: 1.2–3.0) had a higher likelihood of using contraceptives. Urban women had 1.6 times greater odds of using contraceptives compared to their rural counterparts (COR: 0.6; 95% CI: 0.4–0.8).

**Table 3:** Results of logistic regression analyses of contraceptive use for selected background characteristics among currently married women, Sylhet, Bangladesh

Characteristics	Crude OR [95% CI]	Adjusted OR [95% CI]
Current Age (years)		
15–24	Ref	Ref
25–34	2.6*** [2.0,3.5]	1.5 [0.9,2.4]
35–49	1.8*** [1.3,2.5]	0.9 [0.5,1.7]
Number of living children		
≤2	Ref	Ref
3–4	2.5*** [1.9,3.4]	1.8* [1.1,2.7]
≥5	1.9*** [1.5,2.4]	1.6* [1.1,2.2]
Have son		
No son	Ref	Ref
Yes	3.0*** [2.4,3.8]	2.3*** [1.8,2.9]
Ever experience death of a child		
No	Ref	
Yes	1.1 [0.9,1.3]	
Education level of women		
No education	Ref	
Primary	0.8 [0.6,1.2]	
Secondary	0.9 [0.7,1.2]	
Higher	1.0 [0.6,1.7]	
Education of husband		
No education	Ref	Ref
Primary	1.0 [0.8,1.2]	1.1 [0.9,1.5]
Secondary	0.8^1^ [0.6,1.0]	1.0 [0.8,1.4]
Higher	1.3^1^ [0.9,2.1]	1.7* [1.1,2.6]
Working status of women		
No	Ref	Ref
Yes	1.9** [1.2,3.0]	1.6 [1.0,2.6]
Visited by FPW		
No	Ref	Ref
Yes	2.5*** [1.9,3.5]	2.4*** [1.6,3.4]
NGO membership		
No	Ref	Ref
Yes	1.8*** [1.4,2.2]	1.4** [1.1,1.8]
Religion		
Islam	Ref	
Others	1.2 [0.7,1.9]	
Place of residence		
Urban	Ref	Ref
Rural	0.6*** [0.4,0.8]	0.6*** [0.4,0.8]
Wealth quintile		
Poorest	Ref	
Poorer	1.0 [0.7,1.4]	
Middle	1.3 [0.8,2.2]	
Richer	1.2 [0.7,2.1]	
Richest	1.0 [0.6,1.6]	

^1^ *p* < 0.2, *p < 0.05, **p < 0.01, ***p < 0.001, *FPW* family planning worker, *NGO* non-governmental organization

In multivariate analyses, all of the significant predictors in the unadjusted analyses remained significant except age of women (*p* > 0.05). The other notable change was the emergence of higher education level of husbands as a significant predictor of contraceptive use (AOR: 1.7; 95% CI: 1.1–2.6), which was previously not significant in unadjusted analyses (p ~ 0.2).

## Discussion

Modern contraceptive methods were the most common methods used by our study women, and public facilities are the primary source of obtaining contraceptives. We found that various factors simultaneously affect contraceptive use. Our analysis revealed that the number of alive children, presence of a son, husbands’ education level, working status of the women, receiving a visit by an FPW, and NGO membership positively impact contraceptive use while living in a rural area is inversely associated with contraceptives use when adjusted for other factors. We have reconfirmed the significance of these known factors in a new setting.

To reduce TFR to the replacement level (TFR-2.0), family planning programs cannot be successful without societal changes. Though different targets of CPR were set by different programs, none of them were successful due to inadequate societal changes [17]. For example, the Health, Population, and Nutrition Sector Development Program (HPNSDP) previously set a target of achieving CPR by 72% by the year 2016. However, that program did not successfully meet the target due to an inadequate involvement of the society [18]. Family planning needs to be strengthened to address these issues and meet the targeted TFR.

In both rural and urban areas, the public sector is the primary source from which women obtain contraceptives. In addition to strengthening this sector, the government should focus on strengthening contraceptive supply in private sectors as public services are not always able to reach all women throughout the country. Although the contraceptive pill was the most common birth control method, however, long-acting permanent methods (LAPM) as implants, intrauterine devices (IUDs), or sterilizations could be cost-effective for the women in lower socioeconomic statuses or women living in areas that are difficult to access.

Receiving a visit from an FPW within the last six months had the highest positive impact on contraceptive use as women who received the services had more than two times greater odds of using contraceptives than women who did not receive any visit [12, 13, 19]. Women who were visited received advice on which contraceptives were appropriate for them, as well as services from the FPW and ultimately obtaining contraceptives from them. A small proportion of our study women received services from FPWs. Officials planning to scale up such programs should focus on increasing FPW visits to women in order to achieve contraceptive coverage targets. Previous articles from Bangladesh also found that discontinuation of services from FPWs was associated with higher discontinuation of contraceptives﻿' use [19, 20].

We have found a wide variation in prevalence, method choice, and sources of contraceptive use between rural and urban women. In Bangladesh, more than 65% of the people live in rural areas. Thus, public health programs need to prioritize or provide special attention to targeting delivery of services to populations in the countryside [14]. This difference is due to the insufficient health and family planning services available in the rural regions compared to the urban regions, as well as differences in socio-cultural factors between the regions [1]. Moreover, maternal and neonatal mortality rates in rural areas are higher than the urban areas due to limited access to reproductive services and different socio-cultural factors including education level and awareness [2, 21, 22]. Older studies also confirmed these findings [10, 12]. This consistent finding is important for policymakers of Bangladesh as public health services are still not sufficiently strengthened or are under-utilized by a large portion of the people of the predominantly rural regions of the country.

Most studies in the past revealed that employed women tend to have higher use of contraceptives [23, 24]. We also found that women who work outside of their homes have higher contraceptive use rate compared to housewives. Membership in an NGO was found to have a positive impact on the usage of contraceptives. The government could increase the coverage of contraceptive access and use by collaborating with NGOs (such as Grameen Bank, BRAC, or TMSS) and involve women with NGO activities, so women could simultaneously work out of their homes or earn money. This could also increase women empowerment within the household [1].

Similar to previous studies, a family’s desire for a son also had a major impact on contraceptive use [11–13, 23, 25]. We found that the social custom of desiring male children did not change over the study period.

A higher education level of husbands had a positive impact on contraceptives use in our study. We believe that educated husbands have been exposed to public health information or can understand the importance of using contraceptives and can influence their wives to use contraceptives more often than a husband without higher education. This factor is specific for this region in comparison to Bangladesh overall as earlier studies which analyzed the DHS data from Bangladesh did not found this association [11, 13].

Factors which were not significantly associated with contraceptives use in this region also merit discussion. Previous studies from Bangladesh found that highly educated women were more likely to use contraceptives than the women with low level of education [11, 13]. Moreover, education has been described as the best contraceptive as it makes women more aware of the importance of family planning in addition to available work opportunities outside the home [26]. This contrary finding could be due to lack of awareness among all women in the study region about the importance of family planning regardless of their education level. It also indicates that awareness programs should focus on targeting all women of any education level. We did not find any association between religion and contraceptives use, which is another unique finding in our study region compared to other parts of the country [11, 13].

A simultaneous significance of various factors shows that a comprehensive strategy is required for this region of the country in order to achieve the targets of the FP2020. Because only three years remain before the targeted year of 2020, rapid implementation of the findings is crucial.

Our study had several strengths. To our knowledge, this was the first study which looked specifically into the distribution and determinants of contraceptives use in this region of the lowest CPR of Bangladesh. The study sample that was used for our analysis is representative of the whole Sylhet Division, and the sample size was large. The response rate of this survey was also very high (99%) [2].

One limitation of our study is that we were unable to examine several of the factors which were found to be significant in other studies or which might have an association with contraceptives use. For example, one variable collected in our dataset asked whether the woman’s husband was living elsewhere as the reason for not using contraception. However, the data did not record whether the husband lived abroad or within the country, so it was difficult to determine whether intercourse occurred within regular intervals. Also, the analysis included only currently married women, so women who were divorced or widowed but might have sexual exposure were not differentiated. Nevertheless, the proportion of widowed and divorced women was only about 2%, so we did not expect this group to affect our results. Lastly, we analyzed cross-sectional data and only included socioeconomic status of twelve months preceding the survey. Thus causality in our observed associations could not be fully established.

## Conclusions

This study examined representative data for women residing in Sylhet Division using the Bangladesh Demographic and Health Survey (BDHS) of 2014. Our analysis revealed that concerted efforts are required to address multiple factors that affect contraceptive use by married women of reproductive age in Sylhet. From a program planning perspective, it is important to take modifiable factors into account. To scale up the contraceptives campaign in Sylhet Division of Bangladesh and to achieve the targets of the SDGs and FP2020, the government should provide services with FPWs to the women who have less FPW coverage and prioritize women living in rural areas. Successful collaboration between government and NGOs is necessary, and awareness programs should be carried out.

## Abbreviations

AOR: Adjusted odds ratio
BBS: Bangladesh Bureau of Statistics
BDHS: Bangladesh Demographic and Health Survey
CI: Confidence interval
CORs: Crude odds ratio
CPR: Contraceptive prevalence rate
EAs: Enumeration Areas
FP2020: Family Planning 2020
FPW: Family planning worker
HPNSDP: Health, Population, and Nutrition Sector Development Program
IUDs: Intrauterine device
LAPM: Long acting permanent methods
MOHFW: Ministry of Health and Family Welfare
NGO: Non-Government organization
OR: Odds ratio
SDGs: Sustainable Development Goals
TFR: Total Fertility Rate
VIFs: Variance Inflation Factors

## References

[1] United Nations, Department of Economic and Social Affairs, Population Division (2015). World fertility patterns 2015 - data booklet.

[2] National Institute of Population Research and Training (NIPORT), Mitra and Associates, ICF International (2016). Bangladesh demographic and health survey 2014.

[3] Cleland J, Conde-Agudelo A, Peterson H, Ross J, Tsui A (2012). Contraception and health. Lancet.

[4] Ahmed S, Li Q, Liu L, Tsui AO (2012). Maternal deaths averted by contraceptive use: an analysis of 172 countries. Lancet.

[5] Chola L, McGee S, Tugendhaft A, Buchmann E, Hofman K (2015). Scaling Up family planning to reduce maternal and child mortality: the potential costs and benefits of modern contraceptive use in South Africa. PLoS One.

[6] Kost K, Forrest JD, Harlap S (1991). Comparing the health risks and benefits of contraceptive choices. Fam Plann Perspect.

[7] Stover J, Ross J (2010). How increased contraceptive use has reduced maternal mortality. Matern Child Health J.

[8] Stover J, Ross J. How contraceptive use affects maternal mortality. USA: United States agency for international development (USAID); 2008.

[9] United Nations, Sustainable Development Goals. http://www.un.org/sustainabledevelopment/health/. Accessed 02/11/2017.

[10] FAMILY PLANNING 2020. http://www.familyplanning2020.org/entities/70. Accessed 02/11/2017.

[11] Al Kibria GM, Hossen S, Barsha RAA, Sharmeen A, Uddin SI, Paul SK (2016). Factors affecting contraceptive use among married women of reproductive age in Bangladesh. Journal of Molecular Studies and Medicine Research.

[12] Kamal SM (2015). Socioeconomic factors associated with contraceptive use and method choice in urban slums of Bangladesh. Asia Pac J Public Health.

[13] Kamal SM, Islam MA (2010). Contraceptive use: socioeconomic correlates and method choices in rural Bangladesh. Asia Pac J Public Health.

[14] Bangladesh Bureau of Statistics (2011). Bangladesh population and housing census.

[15] Stata Corporation (2013). Stata Statistical Software. Release 13.0.

[16] Maldonado G, Greenland S (1993). Simulation study of confounder-selection strategies. Am J Epidemiol.

[17] Caldwell JC, Barkat-E-Khuda, Caldwell B, Pieris I, Caldwell P (1999). The Bangladesh fertility decline: an interpretation. Popul Dev Rev.

[18] Ministry of Health and Family Welfare (2011). Health, Population and Nutrition Sector Development Program(HPNSDP).

[19] Khan MA (2003). Factors associated with oral contraceptive discontinuation in rural Bangladesh. Health Policy Plan.

[20] Khan MA, Trottier DA, Islam MA (2002). Inconsistent use of oral contraceptives in rural Bangladesh. Contraception.

[21] Kamal S, Ashrafuzzaman M, Nasreen S (2012). Risk factors of neonatal mortality in Bangladesh. Journal of Nepal Paediatric Society.

[22] Kamal SM (2015). What is the association between maternal age and neonatal mortality? An analysis of the 2007 Bangladesh Demographic and Health Survey. Asia Pac J Public Health.

[23] Khan HT (1997). A hierarchical model of contraceptive use in urban and rural Bangladesh. Contraception.

[24] Laskar MS, Mahbub MH, Yokoyama K, Inoue M, Harada N (2006). Factors associated with contraceptive practices of married women in Bangladesh with respect to their employment status. Eur J Contracept Reprod Health Care.

[25] Islam S, Islam MA, Padmadas SS (2010). High fertility regions in Bangladesh: a marriage cohort analysis. J Biosoc Sci.

[26] Carr D (2000). Is education the best contraceptive?.

